# Comparison and identification of serum metabolomic profiles in Stage B and Stage C ejection fraction preserved heart failure

**DOI:** 10.3389/fcvm.2025.1674243

**Published:** 2025-10-07

**Authors:** Song Zou, Li-Wei Zhang, Ting Wang, Yu-Hao Wan, Ke Chai, Si-Ming Wang, Chen Meng, Jian-Ping Cai, Hua Wang, Jie-Fu Yang

**Affiliations:** ^1^ Department of Cardiology, Beijing Hospital, National Center of Gerontology, Institute of Geriatric Medicine, Chinese Academy of Medical Science & Peking Union Medical College, Beijing, China; ^2^ Graduate School of Peking Union Medical College, Chinese Academy of Medical Science, Beijing, China; ^3^ Department of Cardiology, Beijing Hospital, National Center of Gerontology, Institute of Geriatric Medicine, Chinese Academy of Medical Sciences, Beijing, China; ^4^ The Key Laboratory of Geriatrics, Beijing Institute of Geriatrics, Beijing Hospital, National Center of Gerontology, National Health Commission, Institute of Geriatric Medicine, Chinese Academy of Medical Sciences, Beijing, China; ^5^ Peking University Fifth School of Clinical Medicine, Beijing, China

**Keywords:** heart failure with preserved ejection fraction, metabolomics, bioinformatics, pathophysiology, biomarker

## Abstract

**Background:**

Disturbed metabolism correlates with the progression of heart failure with preserved ejection fraction (HFpEF). However, the discrepancy in metabolism between asymptomatic (Stage B) and symptomatic (Stage C) HFpEF patients remains unclear. This study aimed to explore the metabolic differences between Stages B and C HFpEF patients and to screen metabolites to distinguish between the two groups of patients.

**Methods:**

A total of 97 Stage B and 31 Stage C HFpEF patients were included from a previous cohort, named Frailty and Comprehensive Geriatric Assessment in Hospitalized Elderly Patients (registration number: ChiCTR1800017204). Serum metabolites of the participants were identified and quantified using targeted metabolomics (Biocrates MxP® Quant 500 kit).

**Results:**

Differential analysis identified 208 metabolites of 19 categories, of which lipids (*n* = 168), amino acids (*n* = 7), and related metabolites (*n* = 18) accounted for the top three differential metabolites. In addition, the differential metabolites were significantly enriched in 15 metabolic pathways encompassing amino acid metabolism (10 pathways), lipid metabolism (two pathways), carbohydrate metabolism (one pathway), energy metabolism (one pathway), and protein translation (one pathway). Metabolite set enrichment analysis demonstrated that the differential metabolites most likely originated from muscles and were most significantly enriched in renal disease states (continuous ambulatory peritoneal dialysis and chronic renal failure). Three non-heart-specific metabolites, i.e., cystine (AUC = 0.919), stearic acid [FA (18:0), AUC = 0.913], and N-palmitoyl sphingomyelin (SM C 16:0, AUC = 0.898), displayed higher accuracy than N-terminal pro-B-type brain natriuretic peptide (AUC = 0.838) in differentiating Stage B and Stage C patients.

**Conclusion:**

Compared with Stage B control, Stage C patients suffer from extensive metabolic disorders, of which lipid metabolism and amino acid metabolism are mostly significantly impaired. The alterations of metabolites are largely attributed to renal dysfunction and muscle proteolysis. Moreover, non-heart-specific metabolites display potential diagnostic value in differentiating subgroups of patients with HFpEF.

## Introduction

Heart failure (HF) remains a global health challenge, affecting over 56 million individuals worldwide with persistently high mortality rates (1). Epidemiological shifts have established HF with preserved ejection fraction (HFpEF) as the predominant phenotype, constituting >50% of HF cases (2). Despite preserved systolic function, HFpEF carries a prognosis paralleling HF with reduced ejection fraction (HFrEF), with 5-year mortality exceeding 70% (3). This paradox underscores the need to re-evaluate HFpEF pathophysiology beyond ejection fraction-centric paradigms.

The HF continuum progresses through Stages A–D, with Stage B marking asymptomatic cardiac structural abnormalities preceding overt clinical symptoms (4). Alarmingly, Stage B HF affects >40% of community populations (5, 6), of whom 90% exhibit HFpEF (5). This at-risk cohort represents a critical window for intervention to avert progression to Stage C HFpEF—a transition marked by irreversible myocardial remodeling and functional decline (4, 7). However, current management strategies confront two fundamental challenges in Stage B HFpEF: (1) the absence of stage-specific therapies rooted in incomplete understanding of progression mechanisms (4) and (2) the diagnostic inadequacy of heart-derived biomarker, N-terminal pro-B-type brain natriuretic peptide (NT-proBNP), which fails to discriminate Stages B and C due to shared cardiac abnormalities (4). These limitations highlight the urgent need for non-heart-specific biomarkers to differentiate between the two stages.

Emerging evidence positions metabolic dysregulation as a pivotal yet understudied mechanism in HFpEF pathogenesis. Recent clinical trials with metabolic modulators—sodium–glucose cotransporter 2 (SGLT2) inhibitors and glucagon-like peptide-1 receptor agonists (GLP-1 RA)—have demonstrated unprecedented improvements in exercise capacity among symptomatic HFpEF patients (8, 9). These therapeutic breakthroughs suggest that metabolic perturbations may drive disease progression through pathways distinct from traditional hemodynamic models. However, critical knowledge gaps persist in characterizing the metabolic transitions between preclinical (Stage B) and clinical (Stage C) HFpEF—a prerequisite for developing stage-specific interventions.

Notably, current investigations have not comprehensively characterized metabolic alterations across HFpEF disease stages—a crucial omission given the dynamic nature of metabolic adaptations during disease progression. To address these gaps, we employ a targeted metabolomics approach to (1) systematically characterize stage-specific metabolic profiles in HFpEF progression; (2) explore potential metabolic mechanisms promoting HFpEF progression with a bioinformatic approach; and (3) identify candidate metabolic biomarkers with diagnostic potential for distinguishing Stage B and C HFpEF. Our findings may provide novel insights into metabolic drivers of HFpEF advancement and inform the development of stage-specific therapeutic strategies.

## Materials and methods

### Study participants

This study included participants from a previous cohort study, named Frailty and Comprehensive Geriatric Assessment in Hospitalized Elderly Patients (10). The previous study consecutively recruited 1,000 elderly inpatients from September 2018 to February 2019 in Beijing Hospital. That study was approved by the Ethics Committee of Beijing Hospital (approval no. 2018BJYYEC-121-02) and registered at the Chinese Clinical Trial Registry (registration number: ChiCTR1800017204). Written informed consents were obtained from all participants.

The HFpEF diagnosis and HF stages were defined according to the 2022 AHA/ACC/HFSA Guidelines for the Management of Heart Failure (4). Stage C HFpEF patients were those who fulfilled the following criteria: (1) current or previous symptoms or signs of HF; (2) left ventricular ejection fraction (LVEF) ≥ 50%; and (3) evidence of cardiac structural or functional abnormalities. Stage B HFpEF patients were LVEF-preserved (LVEF ≥ 50%) and had cardiac abnormalities but had no current or previous presentation of HF. The criteria for defining cardiac abnormalities (Supplementary Table S1) were proposed previously (11). Patients with cancer, acute infection, rheumatic diseases, hematological disease, acute myocardial infarction, acute cerebral infarction, estimated glomerular filtration rate (eGFR) < 30 mL/(min*1.73 m^2^), abnormal liver function, and LVEF < 50% were excluded. In this study, a total of 97 Stage B and 31 Stage C HFpEF patients were included.

### Metabolomics analysis

Fasting blood samples were collected in the morning after admission. Serum samples were prepared and frozen at −80 °C until further analysis. Metabolite levels were profiled using the Biocrates MxP Quant 500 kit. The details of metabolite extraction and analysis are described elsewhere (12, 13). For metabolite extraction, a 10 μm sample was transferred to a 96-well plate and dried under a nitrogen stream. The samples were then derivatized with 5% phenyl isothiocyanate solution. After incubating in the dark for 1 h, the samples were dried under a nitrogen stream and dissolved in the extraction solvent. The dissolved samples were then mixed and filtered to obtain the extracts. Flow injection analysis-tandem mass spectrometry (FIA-MS/MS) and liquid chromatography-tandem mass spectrometry (LC-MS/MS) methods were used to analyze the extracts. Lipids were analyzed by FIA-MS/MS using a 5500 QTRAP® instrument (AB Sciex, Darmstadt, Germany) with an electrospray ionization source, and non-lipid molecules were analyzed by LC-MS/MS with the same 5500 QTRAP® instrument. MS software (Sciex Analyst®) and Biocrates MetIDQ™ software were used to calculate metabolite concentrations, data assessment, and compilation. Metabolites with levels below the detection limit were excluded from further analyses.

### Statistics

All data were analyzed using GraphPad Prism 8 (GraphPad, San Diego, CA, USA), Microsoft Excel, SPSS 23.0 (IBM Corp., Armonk, NY, USA), R 4.1.2 (Vienna, Austria), and MetaboAnalyst 5.0 (https://www.metaboanalyst.ca). Categorical variables were described as percentages, and normally distributed continuous variables were expressed as mean ± standard deviation. Non-normally distributed continuous data were described as medians (interquartile range: 25th–75th percentiles).

Differences in baseline characteristics between Stage B and C HFpEF participants were tested using the chi-squared test (categorical variables), Mann–Whitney *U* test (non-normally distributed continuous variables), or Student's *t*-test (normally distributed continuous variables). Differential analysis of all metabolites was performed using the Mann–Whitney *U* test, as most metabolites were not normally distributed. The *P*-values across all metabolites within each comparison were adjusted using a false discovery rate (FDR) method to account for multiple testing, and FDR < 0.05 was considered statistically significant. Hierarchical clustering heatmaps were generated using the “pheatmap” package. Kyoto Encyclopedia of Genes and Genomes (KEGG) pathway analysis was performed by the MetaboAnalyst web service with a hypergeometric test for significance and topology analysis for pathway impact (14). Metabolite set enrichment analysis (MSEA) of diseases and origins was also performed using MetaboAnalyst with a hypergeometric test for significance (14). To maximize the identification of differences in metabolic profiles between groups, an orthogonal projection to latent structure-discriminant analysis (OPLS-DA) model was constructed using SIMCA-P 14.1 (Umetrics AB, Sweden). Metabolites with FDR < 0.05 and variable importance in projection (VIP) ≥1.5 were further analyzed with receiver operating characteristic (ROC) curves.

## Results

### Baseline characteristics

The baseline characteristics of patients stratified by Stage B (*n* = 97) and Stage C (*n* = 31) are presented in Table 1. Stage C tend to be older compared with Stage B (80.45 ± 7.41 vs. 77.85 ± 5.95 years, *p* = 0.050), with marked differences observed in cardiac biomarker and renal function. Stage C patients demonstrated significantly elevated NT-proBNP levels (median 700.2 vs. 170.4 pg/mL, *p* < 0.001), lower eGFR [median 63.59 vs. 79.77 mL/(min × 1.73 m^2^), *p* < 0.001], and decreased exercise tolerance. Echocardiographic analysis revealed that Stage C patients had lower LVEF (62% vs. 65%, *p* = 0.001) and higher E wave velocity (0.96 ± 0.28 vs. 0.81 ± 0.22 m/s, *p* = 0.002). Moreover, Stage C patients had larger left atrial dimensions (LAAPD: 42.27 ± 6.11 vs. 38.90 ± 5.37 mm, *p* = 0.004), and more Stage C patients suffered from atrial fibrillation (21.6% vs. 48.4%, *p* = 0.004). No significant differences were observed in gender, body mass index, total cholesterol, total triglyceride, high-density lipoprotein (HDL), low-density lipoprotein (LDL), smoking status, prevalence of hypertension, diabetes, peripheral artery disease, coronary heart disease, valvular disease, and stroke. Medication patterns were generally comparable between groups except fr diuretics (Stage C vs. Stage B: 12.4% vs. 64.5%, *p* < 0.001) and mineralocorticoid receptor antagonists (Stage C vs. Stage B: 0% vs. 12.9%, *p* = 0.003).

**Table 1:** Patients' characteristics.

Characteristics	Stage B (*n* = 97)	Stage C (*n* = 31)	*P*-value
Age (years)	77.85 ± 5.95	80.45 ± 7.41	0.050
Male (%)	48 (49.5%)	10 (32.3%)	0.093
BMI (kg/m^2^)	25.08 ± 3.65	24.51 ± 3.66	0.449
Smoker (%)	28 (28.9%)	6 (19.4%)	0.297
NT-proBNP (pg/mL)	170.4 (89.81–390.6)	700.2 (184.4–1,819)	<0.001
NYHA functional class			<0.001
I (%)	97 (100%)	0 (0%)	
II (%)	0 (0%)	10 (32.3%)	
III (%)	0 (0%)	19 (61.2%)	
IV (%)	0 (0%)	2 (6.5%)	
Disease history			
Atrial fibrillation (%)	21 (21.6%)	15 (48.4%)	0.004
Aortic regurgitation (%)	4 (4.2%)	0 (%)	0.571
Mitral regurgitation (%)	8 (8.2%)	1 (3.2%)	0.687
Hypertension (%)	79 (81.4%)	28 (90.3)	0.245
Diabetes mellitus (%)	34 (35.1%)	14 (45.2%)	0.311
Peripheral artery disease (%)	20 (20.6%)	10 (32.3%)	0.183
Coronary heart disease (%)	52 (53.6%)	18 (58.1%)	0.664
Stroke (%)	32 (33.0%)	7 (22.6%)	0.273
eGFR [mL/(min × 1.73 m^2^)]	79.77 (67.70–89.17)	63.59 (42.81–86.83)	<0.001
Medication			
Diuretics (%)	12 (12.4%)	20 (64.5%)	<0.001
MRAs (%)	0 (0%)	4 (12.9%)	0.003
Antiplatelet drugs (%)	68 (70.1%)	16 (51.6%)	0.059
RAAS inhibitors (%)	43 (44.3%)	10 (32.3%)	0.235
Lipid-lowering drugs (%)	74 (76.3%)	23 (74.2%)	0.813
β-blockers (%)	53 (54.6%)	22 (71.0%)	0.108
Anticoagulants (%)	12 (12.4%)	8 (25.8%)	0.09
Blood lipids			
Total cholesterol (mmol/L)	3.52 (3.10–4.30)	3.89 (3.32–4.40)	0.376
Total triglyceride (mmol/L)	1.12 (0.81–1.53)	1.15 (0.78–1.36)	0.733
HDL (mmol/L)	1.07 (0.92–1.21)	1.01 (0.72–1.12)	0.309
LDL (mmol/L)	2.10 (1.72–2.69)	2.34 (1.68–2.79)	0.336
Echocardiographic parameters			
LVEF (%)	65 (60–65)	62 (60–65)	0.001
LAAPD (mm)	38.90 ± 5.37	42.27 ± 6.11	0.004
E velocity (m/s)	0.81 ± 0.22	0.96 ± 0.28	0.002
A velocity (m/s)	0.94 (0.84–1.1)	1 (0.98–1.2)	0.804
*E*/*A*	0.8 (0.67–1.08)	0.67 (0.61–1.09)	0.837
Septal *e*′ (cm/s)	7 (6–7.2)	7 (6–9)	0.649
Lateral *e*′ (cm/s)	5 (4–6)	5 (5–6)	0.187
Septal *E*/*e*′	12.09 ± 4.66	13.99 ± 5.64	0.171
Lateral *E*/*e*′	16.8 ± 6.2	19.05 ± 10.67	0.265
LV mass/height^2.7^ (g/m^2.7^)	42.81 (37.40–47.63)	46.35 (36.32–51.88)	0.142

BMI, body mass index; NT-proBNP, N-terminal pro-B-type brain natriuretic peptide; NYHA, New York Heart Association; eGFR, estimated glomerular filtration rate; MRAs, mineralocorticoid receptor antagonists; RAAS, renin–angiotensin–aldosterone system; HDL, high-density lipoprotein; LDL, low-density lipoprotein; LV, left ventricular; LVEF, left ventricular ejection fraction; LAAPD, left atrial anteroposterior diameter; *E* velocity, peak early diastolic transmitral flow velocity; *A* velocity, peak late diastolic transmitral flow velocity; septal *e*′, peak early diastolic mitral annular tissue velocity at septal mitral annulus; lateral *e*′ peak early diastolic mitral annular tissue velocity at lateral mitral annulus.

### Differential analysis

Using targeted metabolomics, 630 compounds were identified and quantified, including 107 non-lipid compounds and 523 lipids. After removing compounds with levels below the detection limit, 355 metabolites were statistically analyzed.

**Figure 1:**
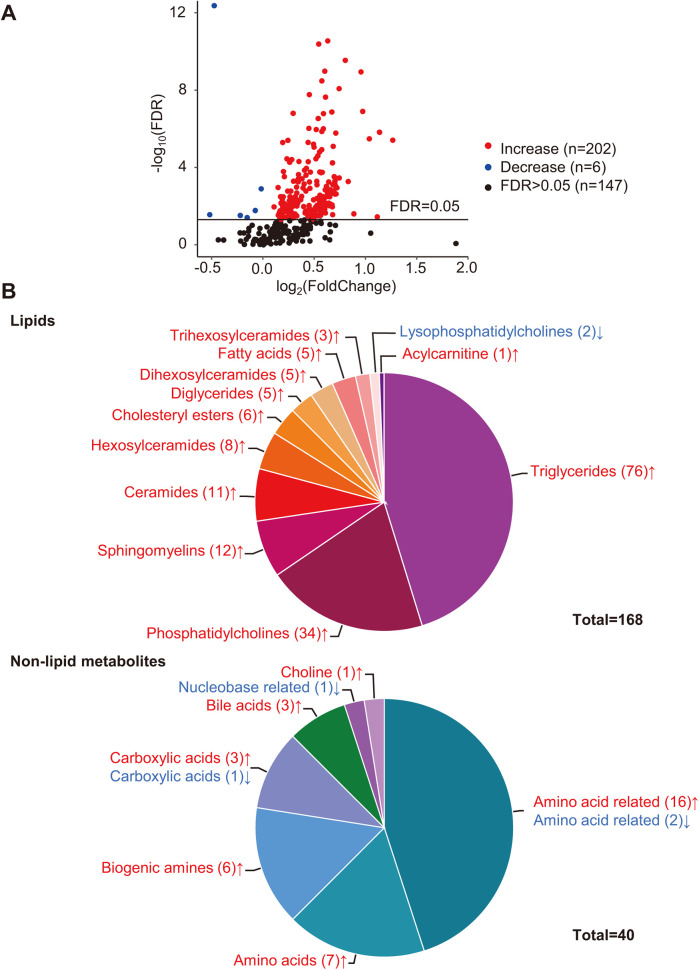
Differential analysis of metabolites. **(A)** Volcano plot of quantified metabolites in Stage C vs. Stage B patients. Each dot represents a metabolite. *Y*-axis: significance plotted as -log10 FDR (false discovery rate) with a cutoff of 0.05 (horizontal line). *X*-axis: effect size plotted as log2 fold change. Red dots represent increased metabolites, blue dots represent decreased metabolites, and black dots represent metabolites that were not significantly changed. **(B)** Categories of differential metabolites. Each part with a different color in the circular ring represents each category of metabolites. The area of each part represents the proportion of metabolites of each category to all differential metabolites; red font (↑) and blue font (↓) indicate metabolites that were significantly increased or decreased, respectively.

Differential analysis identified 208 metabolites in 19 categories that differed significantly (FDR < 0.05) between the two groups (Figures 1A,B). Detailed concentrations of the 208 differential metabolites are summarized in Supplementary Table S2. Compared with the Stage B group, almost all differential metabolites (202/208) significantly increased in symptomatic patients, and only six metabolites significantly decreased (Figure 1B and Supplementary Table S2).

Lipids accounted for the largest proportion of differential metabolites (168/208). The differential lipids include triglycerides (TGs, *n* = 76), diglycerides (DGs, *n* = 5), fatty acids (FAs, *n* = 5), cholesteryl esters (CEs, *n* = 6), phosphatidylcholines (PCs, *n* = 34), lysophosphatidylcholines (LysoPCs, *n* = 2), acylcarnitine (Acar, *n* = 1), ceramides (Cers, *n* = 11) and their derivates, hexosylceramides (Hexcers, *n* = 8), dihexosylceramides (Hex2Cers, *n* = 5), trihexosylceramides (Hex3Cers, *n* = 3), and sphingomyelins (SMs, *n* = 12) (Figure 1B). The Pearson correlation coefficients among the differential lipids with similar structures, such as TGs–DGs, PCs, and Cers, tended to gather in the hierarchical clustering heatmap (Supplementary Figure S1).

Among the non-lipid metabolites, amino acids (AAs) and AA-related metabolites accounted for the top two differential metabolites, with 23 compounds (7 AAs and 16 AA-related metabolites) significantly increased and only two AA-related metabolites, carnosine and phenylacetylglycine, significantly decreased (Figure 1B and Supplementary Table S2). In addition, six biogenic amines, three carboxylic acids, three bile acids, and choline significantly increased (Figure 1B and Supplementary Table S2). One carboxylic acid (succinic acid) and one nucleobase-related metabolite (hypoxanthine) significantly decreased (Figure 1B and Supplementary Table S2).

### Pathway analysis and enrichment analysis of differential metabolites

**Figure 2:**
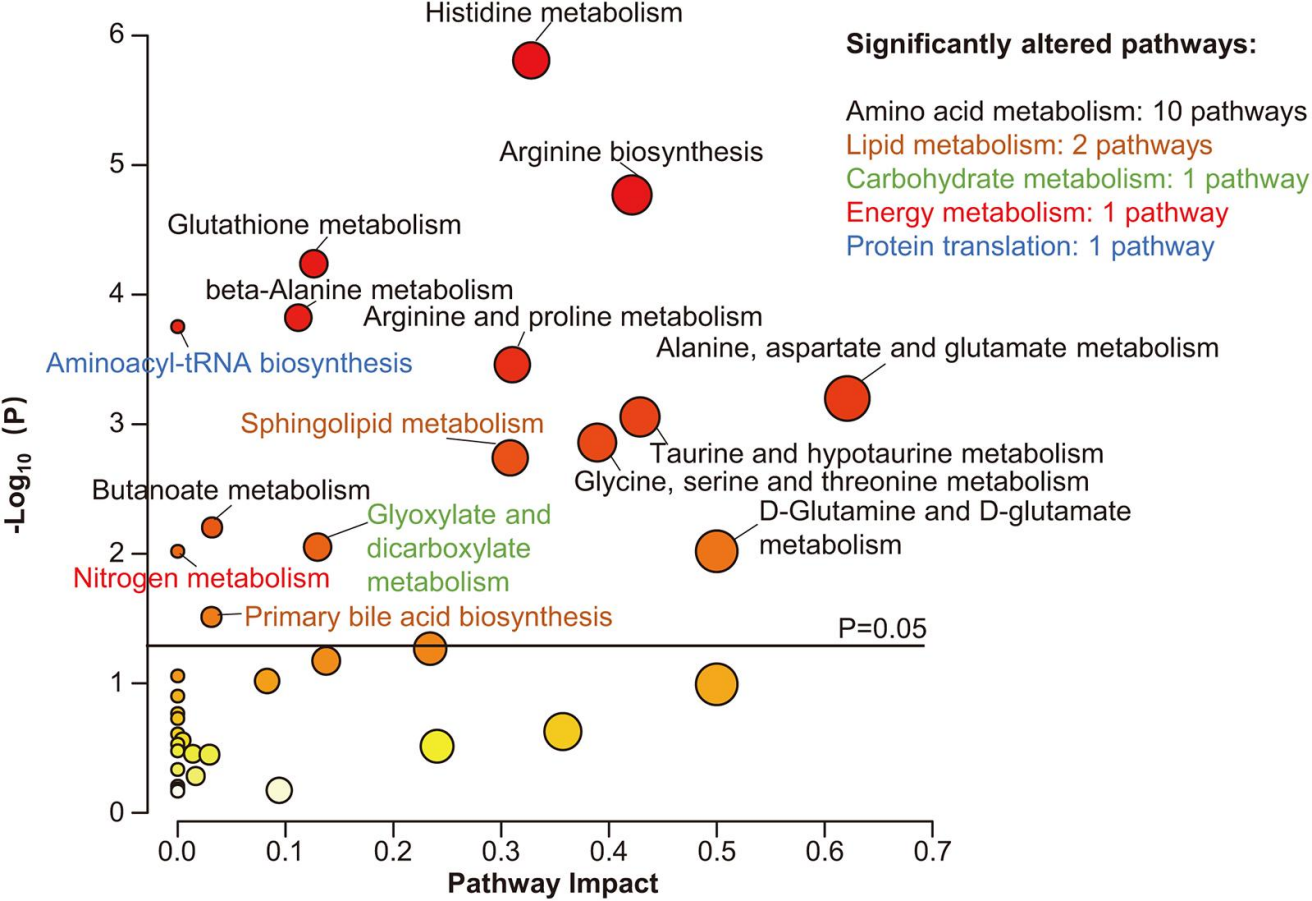
Pathway analysis of differential metabolites. Scatter plot presenting enriched metabolic pathways. The color gradient indicates the significance of the pathway ranked by *P*-value (yellow, higher *P*-values; red, lower *P*-values), and the circle's size indicates the pathway impact score (the larger the circle, the higher the impact score). Significantly enriched pathways were marked by names, and the colors of the names represent categories of enriched pathways. The black horizontal line indicates *P* = 0.05.

To investigate the metabolic pathways related to the metabolic differences between Stage B and C patients, KEGG pathway analysis was performed using the MetaboAnalyst web service. Altogether, 38 metabolic pathways were mapped, of which 15 were significantly enriched, most of which were involved in AA metabolism (10/15) (*P* < 0.05, Figure 2). In addition, pathways concerning lipid metabolism (2/15), carbohydrate metabolism (1/15), energy metabolism (1/15), and protein translation (1/15), although impacted relatively lower by differential metabolites, were also significantly changed (Figure 2). As shown in Figure 2, the top three significantly altered pathways were the histidine, arginine, and glutathione metabolic pathways.

**Figure 3:**
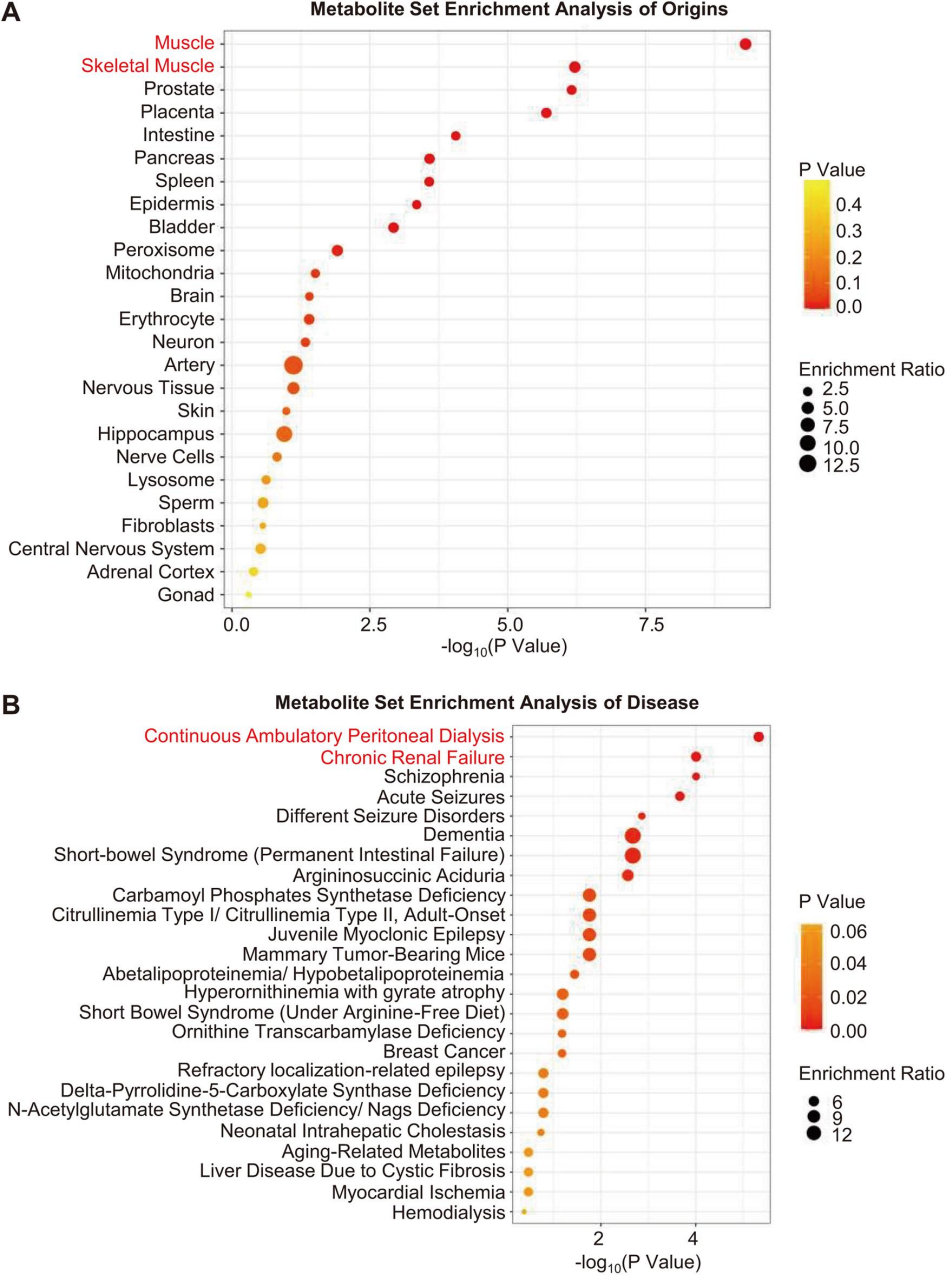
Metabolite set enrichment analysis. **(A,B)** Enrichment analysis of origins and associated diseases of differential metabolites. The color gradient indicates the significance of the enriched organs or diseases ranked by *P*-value (yellow, higher *P*-values; red, lower *P*-values), and the circle's size indicates the enrichment ratio (the larger the circle, the higher the enrichment ratio). Red font indicates organs or diseases that were most significantly enriched.

MSEA revealed that muscles were the most likely sources of differential metabolites (Figure 3A). Moreover, based on MSEA, differential metabolites were most significantly enriched in renal disease states, including continuous ambulatory peritoneal dialysis and chronic renal failure (Figure 3B).

### Diagnostic value of metabolic biomarkers

**Figure 4:**
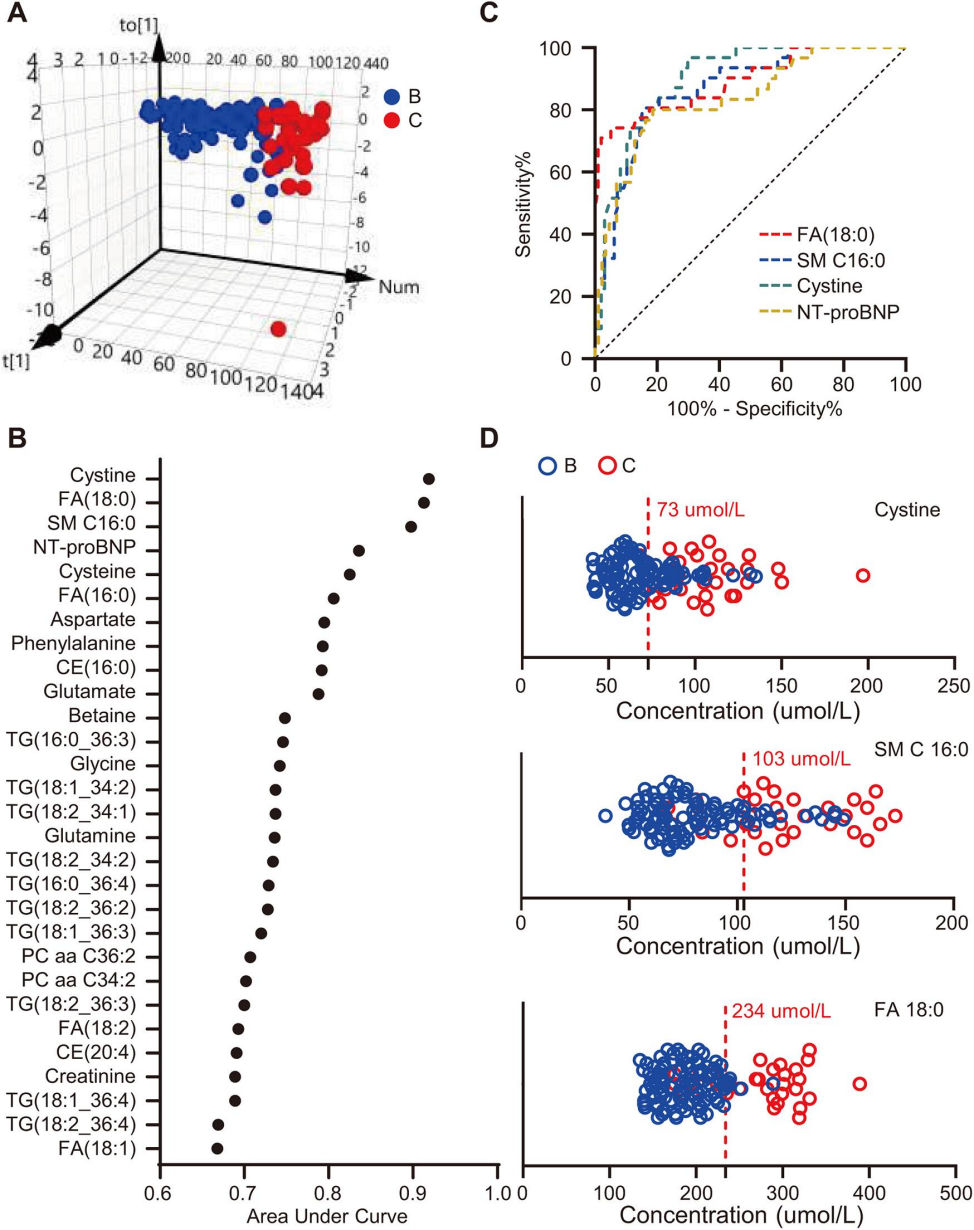
Screening metabolites with potential diagnostic value. **(A)** Three-dimensional scatter plot of OPLS-DA model analysis for Stage C patients (red dot) vs. Stage B patients (blue dot) by the main components. **(B)** Plot of the area under the curve of each metabolite to differentiate Stage B and Stage C patients. **(C)** Receiver operating characteristic curves of FA (18:0), SM C 16:0, cystine, and NT-proBNP. **(D)** Concentrations and optimal cutoff values of FA (18:0), SM C 16:0, and cystine to differentiate Stage B and C patients.

To screen metabolites that maximally contributed to the separation of HFpEF stages, an OPLS-DA model was created based on 355 metabolites that passed quality control. OPLS-DA plot demonstrated clear separation between Stage B and Stage C patients (Figure 4A). The goodness-of-fit parameters (R2X = 0.756, R2Y = 0.67) revealed a good fit, and the parameter for prediction (Q2 = 0.444) suggested a moderate predictive ability of this model. The R2 and Q2 interceptive values were 0.273 and −0.387, respectively, after 200 random permutations, suggesting no overfitting. The criteria to screen potential biomarkers for discriminating preclinical and clinical HFpEF patients were VIP > 1.5 and FDR < 0.05. Of the 355 qualified metabolites, 28 fulfilled these criteria (Supplementary Table S3). We further assessed the diagnostic accuracy of the 28 metabolites using ROC curves. We found that cystine, FA (18:0), and SM C 16:0 were the metabolites with the highest areas under the curves (AUC = 0.919, 0.913, and 0.898, respectively), which were even higher than those of NT-proBNP (AUC = 0.838) (Figures 4B,C). Moreover, their combinations with NT-proBNP further improved the diagnostic accuracy. The AUCs were 0.924, 0.969, and 0.937 for NT-proBNP plus FA (18:0), NT-proBNP plus SM C 16:0, and NT-proBNP plus cystine, respectively. Based on the maximum Youden's index (maximal sum of sensitivity and specificity), the optimal cutoff concentrations of cystine, SM C 16:0, and FA (18:0) were 73, 103, and 234 µmol/L, respectively (Figure 4D).

## Discussion

This study explored the metabolic differences between patients with Stage B and Stage C HFpEF. Compared to the Stage B group, multiple categories of metabolites and corresponding pathways, especially lipids and AAs, were significantly altered in Stage C patients. Bioinformatics analysis revealed that the differential metabolites most likely originated from muscles and were most significantly enriched in renal disease states. Three non-heart-specific metabolites, cystine, SM C 16:0, and FA (18:0), differentiated between preclinical and clinical HFpEF patients with accuracy higher than NT-proBNP.

### Pathophysiological significance

#### Impaired lipid metabolism and abnormal energetic metabolism

Lipid metabolism is strongly associated with cardiovascular diseases. A high-fat diet has previously been reported to induce cardiac abnormalities and murine HFpEF (15, 16). Mechanistically, the accumulation of lipids, including TGs (17, 18), CEs (19), PCs (20), Cers (21, 22), and glycosylated Cers (17), in the myocardium results in cardiac structural and functional abnormalities. Thus, we speculate that the excessively increased serum lipids in Stage C patients account for their worse cardiac parameters (Figure 1B and Table 1).

We also observed that lipids in the same categories, especially TGs–DGs, PCs, FAs, LysoPCs, Cers, and HexCers, tend to cluster together in the hierarchical clustering heatmap (Supplementary Figure S1), suggesting a strong intrinsic association between lipids in the same class. Theoretically, this association should be attributed to shared metabolic pathways for the same class of lipids. These results suggest that intrinsic metabolic defects are the cause of disturbed lipid metabolism in Stage C patients. Consistent with our results, previous investigations also reported that fatty acid metabolism is impaired in HFpEF patients' or animals' myocardium (23, 24), and restoring fatty acid metabolism corrected HFpEF phenotype in animals (25–27).

Lipids are the storage form of energy. Recently, two glucose-lowering drugs, SGLT2 inhibitor and GLP-1RA, were reported to improve the exercise tolerance of symptomatic HFpEF patients (8, 9). Moreover, a previous study reported that exercise training reduced Stage B HFpEF patients' LV myocardial stiffness (7). These investigations, together with our findings, provide a possibility that decreasing energy, whether by drugs or exercise, helps to prevent Stage B HFpEF progression.

In this research, blood lipids tested by the hospital's clinical laboratory, including total cholesterol, total triglyceride, HDL, and LDL, were not significantly different between the two groups (Table 1). However, the metabolomics approach identified various significantly altered lipids (Figure 1 and Supplementary Table S2). This may be owing to the more precise detectability of the metabolomics approach, which can detect differences at the molecular level.

#### Amino acid metabolism disorder and HFpEF

As shown in Figure 2, the differential metabolites were significantly enriched in 10 AA metabolism pathways with high impact. AAs, AA-related metabolites, and biogenic amines are metabolically related. Consistently, previous studies also demonstrate impaired AA metabolism in HFpEF (23, 28, 29). In this study, we speculate that muscular abnormalities and decreased renal function partly account for the perturbation of AA metabolism.

In this study, MSEA revealed that muscles were the most significantly enriched origins of differential metabolites (Figure 3A). Seven essential AAs, including glutamine, glycine, phenylalanine, glutamate, aspartate, arginine, and cysteine, which participate in protein biosynthesis, increased in the serum of Stage C patients (Figure 1B and Supplementary Table S2). Moreover, the levels of two muscle-related metabolites, sarcosine (30) and taurine (31), also increased in the serum of patients with Stage C disease (Supplementary Table S2). These results suggest that Stage C patients suffer from increased muscle proteolysis, a process of releasing AAs into the blood, which also accounts for the decreased exercise tolerance of Stage C patients. Similarly, previous studies also reported increased proteolysis in both the myocardium (32) and skeletal muscles of HFpEF patients (33).

In addition, differential metabolites were most significantly enriched in renal disease states (Figure 3B), and Stage C patients had lower eGFR (Table 1). Creatinine, kynurenine, asymmetric dimethylarginine (ADMA), symmetric dimethylarginine (SDMA), and homocysteine are uremic toxins (34, 35), and their increase is accompanied by a decline in eGFR (Supplementary Table S2 and Table 1). In addition, ADMA and SDMA can decrease nitric oxide (NO) biosynthesis by inhibiting NO synthase and suppressing the transportation of arginine, respectively (36, 37). Moreover, impaired NO bioavailability contributes to cardiac dysfunction in HFpEF patients (38). Our results provided a possible mechanistic link between the decline of renal function and the exacerbation of HFpEF—that is, decreased renal function in HFpEF patients increases serum uremic toxins, which impair NO biosynthesis and exacerbate cardiac dysfunction. Future study would concentrate on whether and how slowing down eGFR decline or clearing uremic toxins (such as ADMA and SDMA) helps to prevent Stage B HFpEF progression.

### Diagnostic value of serum metabolites

In this study, we found that three non-heart-specific metabolites, i.e., FA (18:0), SM C 16:0, and cystine, were more accurate than NT-proBNP in differentiating between patients with Stage B and Stage C HFpEF (Figure 4B). Although FA (18:0), SM C 16:0, and cystine are not heart-specific metabolites, their high diagnostic accuracy should be ascribed to systemic changes that occur during the progression of HFpEF (39, 40). In addition, the accuracy of NT-proBNP is limited as it also increases in Stage B patients (4). In summary, these findings reveal the potential value of non-heart-specific biomarkers for differentiating the subtypes of cardiovascular disease.

### Limitations

In this research, patients' baseline characteristics, including age and comorbidities, were significantly different between the two groups. Stage C patients were older, more vulnerable to atrial fibrillation, had lower eGFR, and worse cardiac function (Table 1), which may impact the metabolome to some extent. As a matter of fact, metabolites associated with renal dysfunction, such as ADMA and SDMA (34), did increase in Stage C group serum, which means the kidney-related metabolites may provide a mechanistic link between renal dysfunction and HFpEF progression. Aging is a risk factor of HFpEF, which suggests that aging may promote the progression of HFpEF via an altered metabolome. Thus, it is reasonable that Stage C patients are older than Stage B control. Moreover, SGLT2 inhibitors were not prescribed for Stage C patients as they were enrolled from 2018 to 2019 when this drug had not been recommended by guidelines. Future studies must include patients taking SGLT2 inhibitors to clarify the residual risk under such therapy. In addition, it would have been valuable to take healthy subjects as controls. However, it was not available as all participants were inpatients in our previous cohort. Finally, the findings of this study were derived from a single cohort. Therefore, the results should be interpreted with caution.

## Conclusion

The metabolic differences between Stage B and Stage C HFpEF patients are diverse, involving various metabolites and metabolic pathways, among which lipid metabolism and AA metabolism are most significantly impaired. The perturbations of metabolism are largely attributed to muscle proteolysis and renal dysfunction. Non-heart-specific metabolites, including FA (18:0), SM C 16:0, and cystine, are candidate biomarkers to differentiate HFpEF subgroups with accuracy higher than NT-proBNP. In the future, therapeutic strategies targeting excessive energy, renal dysfunction, and uremic toxins have the potential to prevent Stage B patients from progression.

## Data Availability

The original contributions presented in the study are included in the article/Supplementary Material, further inquiries can be directed to the corresponding author.
