# Supplementary material for: Comparison and identification of serum metabolomic profiles in Stage B and Stage C ejection fraction preserved heart failure
Source: Front Cardiovasc Med. 2025 Oct 7;12:1674243. doi: 10.3389/fcvm.2025.1674243 (PMC12537769; doi:10.3389/fcvm.2025.1674243)
Supplement: Supplementary file 1 [file Datasheet1.pdf]

Comparison And Identification of Metabolomic Profiles in Stage B And Stage C Ejection Fraction Preserved Heart Failure

Supplementary Tables

Table S1. Diagnostic Criteria of Cardiac Abnormalities.

Table S2. Serum Concentration of Differential Metabolites.

Table S3. Metabolites with FDR<0.05 and VIP>1.5.

Supplementary Figure(s)

Figure S1. Hierarchical Clustering Heatmap of Pearson Correlation Coefficient of Differential Lipids. In this clustering heatmap, lipids with similar correlation coefficients were clustered together. TGs, Triglycerides; DGs, Diglycerides; FAs, Fatty acids; CEs, Cholesteryl esters; Cers, ceramides; HexCers, hexosylceramides; Hex2Cer, dihexosylceramides; Hex3Cer, trihexosylceramides; SMs, sphingomyelins; PCs, Phosphatidylcholines; LysoPCs, lysophosphatidylcholine; Acar, acylcarnitine.

Table S1. Diagnostic Criteria of Cardiac Abnormalities

	Male	Female
Valvular heart disease	Moderate or greater stenosis or regurgitation in the aortic or mitral valve;	
NT-proBNP	NT-proBNP >125pg/ml	
LV enlargement	LVEDV/BSA >60.2 mL/m^2 (men);	LVEDV/BSA >51.9 mL/m^2 (women);
LV hypertrophy	LV mass/height$^{2.7}$ >45 g/m$^{2.7}$ (men);	LV mass/height$^{2.7}$ >41.5 g/m$^{2.7}$ (women);
Diastolic dysfunction	Septal e′ <4.3 cm/s (men);	Septal e′ <4.1 cm/s (women);
	Septal E/e′ ratio >14.8 (men);	Septal E/e′ ratio >17.4 (women);
	LAAPD >39.2 mm (men aged 65–69 years);	LAAPD >38.3 mm (women aged 65–69 years);
	LAAPD >40.3 mm (men aged ≥70 years);	LAAPD >38.6 mm (women aged ≥ 70 years);
Systolic dysfunction	LVEF<54.6% (aged 65-69 years)	LVEF<54.5% (aged 65-69 years)

LVEF<53% (aged ≥ 70 years)	LVEF<53.5% (aged ≥ 70 years)

Abbreviation: NT-proBNP, N-terminus pro-brain natriuretic peptide; LVEDV, Left ventricular end-diastolic volume; BSA, Body surface area; LV, Left Ventricular; LAAPD, Left atrial anteroposterior diameter; LVEF, Left ventricular ejection fraction

Table S2. Serum Concentrations of Differential Metabolites

Metabolites	Categories	Serum Concentration (umol/L)		Foldchange	Change Direction	FDR
		Stage B (n=97)	Stage C (n=31)			
TG(16:0_36:3)	Triglycerides	159 (116-246)	252 (189-351)	1.63	Increase	0.0005
TG(18:1_34:2)	Triglycerides	152 (115-231)	249 (174-347)	1.63	Increase	0.0008
TG(18:2_34:1)	Triglycerides	154 (113-236)	245 (175-327.5)	1.61	Increase	0.0008
TG(18:2_34:2)	Triglycerides	161 (109-222)	260 (161-318.5)	1.62	Increase	0.0007
TG(18:1_36:3)	Triglycerides	114 (90.1-174)	178 (121-258)	1.55	Increase	0.0023
TG(16:0_36:4)	Triglycerides	89.9 (61.4-129)	143 (94.35-178.5)	1.63	Increase	0.0009
TG(18:2_36:2)	Triglycerides	72.9 (53.9-107)	118 (82.2-162)	1.58	Increase	0.0014
TG(18:2_36:3)	Triglycerides	85 (58.8-111)	109 (82.35-170)	1.56	Increase	0.0071
TG(18:1_36:4)	Triglycerides	55.5 (38.1-72.8)	73.7 (53-113.5)	1.52	Increase	0.0108
TG(18:2_36:4)	Triglycerides	32.4 (20.8-45.7)	42.6 (27.3-71.05)	1.53	Increase	0.0227
TG(18:1_34:3)	Triglycerides	28 (19.6-38.8)	38.2 (26.85-57.1)	1.46	Increase	0.0149

TG(18:2_32:0)	Triglycerides	16.3 (10.4-23.6)	27.5 (19.35-37.65)	1.78	Increase	0.0005
TG(16:1_36:3)	Triglycerides	24.6 (17.2-32.3)	32 (20.25-45.7)	1.42	Increase	0.0356
TG(18:2_34:3)	Triglycerides	23.6 (16.4-32.6)	32 (19.35-46.15)	1.45	Increase	0.0322
TG(18:3_34:1)	Triglycerides	13.1 (9.97-18.7)	19.4 (13.3-28.75)	1.50	Increase	0.0108
TG(18:2_32:1)	Triglycerides	16.3 (12-23.8)	22.7 (17.4-36.4)	1.48	Increase	0.0190
TG(18:1_32:1)	Triglycerides	18.8 (12.4-28.9)	27.4 (17.3-43.3)	1.42	Increase	0.0278
TG(20:4_36:2)	Triglycerides	6.61 (5.25-9.52)	9.6 (7.35-12.85)	1.53	Increase	0.0033
TG(18:0_36:4)	Triglycerides	7.79 (5.22-11)	12.1 (7.81-17.5)	1.60	Increase	0.0086
TG(18:3_34:2)	Triglycerides	11.1 (7.97-16.9)	16.5 (10.5-23.7)	1.48	Increase	0.0295
TG(16:0_34:3)	Triglycerides	10.3 (6.95-15.1)	17.1 (10.135-22)	1.52	Increase	0.0086
TG(20:4_36:3)	Triglycerides	5.92 (4.57-7.72)	7.76 (6.29-10.95)	1.47	Increase	0.0028
TG(16:1_34:2)	Triglycerides	9.43 (6.41-13.3)	14 (8.805-20.45)	1.52	Increase	0.0138
TG(16:1_34:1)	Triglycerides	8.73 (6.06-14.2)	14.1 (9.29-21.75)	1.52	Increase	0.0095
TG(20:4_34:2)	Triglycerides	6.62 (4.58-8.88)	9.38 (6.37-13.6)	1.47	Increase	0.0058
TG(20:4_34:1)	Triglycerides	6.24 (4.08-9.28)	8.73 (6.12-14.1)	1.46	Increase	0.0109

TG(16:0_36:5)	Triglycerides	7.16 (4.91-10.2)	11 (6.23-15.55)	1.51	Increase	0.0141
TG(18:2_38:4)	Triglycerides	3.02 (2.35-4.05)	4.42 (3.205-6.565)	1.64	Increase	0.0024
TG(18:1_38:5)	Triglycerides	3.95 (3.02-5.24)	5.32 (4.515-7.165)	1.44	Increase	0.0093
TG(22:5_34:1)	Triglycerides	2.42 (1.56-3.85)	3.77 (2.725-5.95)	1.65	Increase	0.0011
TG(20:4_36:4)	Triglycerides	2.58 (1.77-3.47)	3.66 (2.69-5.805)	1.54	Increase	0.0010
TG(18:2_35:2)	Triglycerides	2.95 (2.15-4.67)	4.88 (3.155-6.14)	1.52	Increase	0.0015
TG(22:6_34:2)	Triglycerides	4.05 (2.37-6.81)	5.83 (3.81-9.355)	1.60	Increase	0.0275
TG(22:5_34:2)	Triglycerides	3 (2.08-4.17)	4.24 (2.775-6.54)	1.53	Increase	0.0093
TG(18:2_38:5)	Triglycerides	3.89 (2.91-5.12)	4.94 (3.49-6.96)	1.42	Increase	0.0184
TG(16:0_38:3)	Triglycerides	2.4 (1.53-3.75)	4.16 (2.91-5.515)	1.61	Increase	0.0007
TG(18:1_35:2)	Triglycerides	3.25 (2.43-4.89)	4.75 (3.43-6.46)	1.43	Increase	0.0120
TG(18:2_38:6)	Triglycerides	2.14 (1.54-3.1)	3.31 (2.125-4.93)	1.61	Increase	0.0017
TG(16:0_38:4)	Triglycerides	3.37 (2.09-4.56)	4.22 (3.315-6.94)	1.47	Increase	0.0108
TG(18:2_33:1)	Triglycerides	2.6 (1.8-3.58)	3.83 (2.81-4.91)	1.49	Increase	0.0058

TG(17:0_36:3)	Triglycerides	2.49 (1.73-4.02)	3.71 (2.755-4.855)	1.45	Increase	0.0108
TG(18:2_33:2)	Triglycerides	2 (1.27-2.8)	3.23 (2.17-3.83)	1.56	Increase	0.0006
TG(16:0_38:5)	Triglycerides	3.09 (2.2-4.26)	4.26 (3.115-5.915)	1.43	Increase	0.0152
TG(16:0_38:6)	Triglycerides	1.98 (1.4-2.86)	2.96 (2.03-4.005)	1.51	Increase	0.0054
TG(16:0_40:6)	Triglycerides	1.74 (1.13-2.53)	2.72 (1.645-3.56)	1.54	Increase	0.0086
TG(18:1_38:6)	Triglycerides	2.52 (1.96-3.38)	3.38 (2.39-4.145)	1.38	Increase	0.0351
TG(18:1_33:2)	Triglycerides	1.99 (1.34-2.69)	2.85 (2.06-3.46)	1.44	Increase	0.0040
TG(18:3_36:1)	Triglycerides	1.7 (1.33-2.28)	2.54 (1.675-3.77)	1.47	Increase	0.0276
TG(20:3_34:2)	Triglycerides	2.46 (1.74-3.4)	3.15 (2.38-4.72)	1.42	Increase	0.0277
TG(20:2_34:2)	Triglycerides	1.7 (1.23-2.38)	2.5 (1.83-3.48)	1.50	Increase	0.0032
TG(20:3_34:1)	Triglycerides	2.28 (1.49-3.49)	2.96 (2.065-4.82)	1.41	Increase	0.0446
TG(16:0_35:2)	Triglycerides	1.48 (0.936-2.13)	2.09 (1.535-3.335)	1.57	Increase	0.0033
TG(18:0_38:6)	Triglycerides	1.1 (0.809-1.52)	1.69 (1.17-2.33)	1.57	Increase	0.0075
TG(22:4_34:2)	Triglycerides	0.802 (0.535-1.14)	1.27 (0.8625-1.96)	1.67	Increase	0.0003
TG(20:3_36:3)	Triglycerides	1.92 (1.37-2.38)	2.43 (1.435-3.295)	1.41	Increase	0.0495

TG(17:1_36:3)	Triglycerides	1.35 (1.06-1.93)	2.09 (1.455-2.915)	1.46	Increase	0.0041
TG(18:3_34:0)	Triglycerides	1.36 (0.967-1.84)	2.15 (1.395-2.88)	1.52	Increase	0.0068
TG(16:0_38:2)	Triglycerides	0.946 (0.608-1.48)	1.48 (1.075-2.29)	1.57	Increase	0.0017
TG(17:0_36:4)	Triglycerides	1.39 (0.969-2.13)	2.13 (1.46-2.465)	1.38	Increase	0.0150
TG(18:1_35:3)	Triglycerides	1.41 (1.02-1.95)	1.98 (1.4-2.545)	1.36	Increase	0.0152
TG(16:1_36:1)	Triglycerides	1.75 (1.24-2.62)	2.38 (1.675-3.71)	1.37	Increase	0.0490
TG(18:0_36:5)	Triglycerides	0.723 (0.491-1.1)	1.16 (0.669-1.7)	1.57	Increase	0.0096
TG(20:0_32:4)	Triglycerides	0.722 (0.454-1.02)	1.02 (0.735-1.42)	1.63	Increase	0.0024
TG(18:3_32:0)	Triglycerides	1.07 (0.721-1.73)	1.65 (1-2.51)	1.52	Increase	0.0170
TG(16:0_35:3)	Triglycerides	0.915 (0.644-1.34)	1.46 (1.045-1.68)	1.48	Increase	0.0079
TG(20:4_34:0)	Triglycerides	0.719 (0.482-1.06)	1.04 (0.7285-1.745)	1.47	Increase	0.0102
TG(17:0_34:2)	Triglycerides	0.797 (0.527-1.16)	1.01 (0.7425-1.82)	1.51	Increase	0.0117
TG(20:4_32:0)	Triglycerides	0.666 (0.368-0.985)	0.923 (0.632-1.76)	1.46	Increase	0.0092
TG(16:1_38:4)	Triglycerides	0.621 (0.429-0.777)	0.815 (0.506-1.215)	1.44	Increase	0.0310

TG(20:0_32:3)	Triglycerides	0.423 (0.307-0.614)	0.752 (0.454-0.939)	1.59	Increase	0.0023
TG(16:0_34:4)	Triglycerides	0.924 (0.623-1.43)	1.44 (0.779-1.82)	1.40	Increase	0.0396
TG(16:1_34:0)	Triglycerides	0.978 (0.602-1.6)	1.36 (0.8715-2.22)	1.36	Increase	0.0323
TG(16:0_33:2)	Triglycerides	0.481 (0.299-0.724)	0.695 (0.4835-1.06)	1.50	Increase	0.0130
TG(20:3_34:0)	Triglycerides	0.247 (0.168-0.376)	0.356 (0.257-0.553)	1.48	Increase	0.0070
TG(22:5_32:1)	Triglycerides	0.303 (0.191-0.451)	0.41 (0.292-0.697)	1.35	Increase	0.0276
TG(20:3_32:0)	Triglycerides	0.317 (0.196-0.46)	0.386 (0.3045-0.7015)	1.40	Increase	0.0229
DG(18:1_18:2)	Diglycerides	9.92 (6.87-11.7)	12.3 (9.15-15.45)	1.37	Increase	0.0094
DG(18:2_18:2)	Diglycerides	8.56 (6.15-11.4)	10.4 (8.16-14.45)	1.33	Increase	0.0191
DG(18:1_18:1)	Diglycerides	2.17 (1.68-2.97)	2.79 (2.12-3.665)	1.33	Increase	0.0224
DG(16:0_18:1)	Diglycerides	2.14 (1.76-2.9)	2.58 (2.185-3.55)	1.26	Increase	0.0323
DG(16:0_18:2)	Diglycerides	2.49 (1.84-3.09)	2.86 (2.485-3.255)	1.23	Increase	0.0407
FA(16:0)	Fatty acids	299 (270-325)	345 (322.5-386.5)	1.18	Increase	0.0000
FA(18:0)	Fatty acids	187 (161-209)	289 (229.5-315)	1.46	Increase	0.0000
FA(18:2)	Fatty acids	179 (140-214)	215 (177.5-246)	1.23	Increase	0.0047

FA(18:1)	Fatty acids	123 (97.9-149)	136 (122.5-163)	1.18	Increase	0.0134
FA(20:1)	Fatty acids	2.75 (2.17-4.86)	6.84 (5.655-8.04)	1.94	Increase	0.0000
CE(20:4)	Cholesteryl esters	242 (186-319)	307 (246.5-374.5)	1.26	Increase	0.0098
CE(16:0)	Cholesteryl esters	155 (122-214)	216 (188.5-276)	1.34	Increase	0.0001
CE(22:6)	Cholesteryl esters	33.6 (25-44.8)	45.2 (35.3-50.7)	1.25	Increase	0.0054
CE(18:3)	Cholesteryl esters	33.6 (24.3-50.2)	41 (32.4-53.55)	1.22	Increase	0.0364
CE(18:0)	Cholesteryl esters	10.2 (8.11-13.4)	12.5 (10.6-15.55)	1.20	Increase	0.0112
CE(22:5)	Cholesteryl esters	2.03 (1.57-3)	3.03 (2.33-4.3)	1.44	Increase	0.0008
SM C16:0	Sphingomyelins	74.9 (63.4-90.3)	118 (103.5-146.5)	1.52	Increase	0.0000
SM C16:1	Sphingomyelins	11.3 (9.46-13.4)	17.1 (15.15-19.8)	1.49	Increase	0.0000
SM C18:0	Sphingomyelins	10.2 (8.56-13.7)	14.3 (12.8-19.75)	1.41	Increase	0.0000
SM C18:1	Sphingomyelins	6.36 (4.82-8.15)	7.95 (7.31-9.945)	1.34	Increase	0.0003
SM (OH) C14:1	Sphingomyelins	2.58 (2.04-3.21)	3.66 (3.145-4.38)	1.43	Increase	0.0000
SM C24:0	Sphingomyelins	10.8 (8.69-13)	13.3 (11.65-15.15)	1.23	Increase	0.0005

SM (OH) C22:1	Sphingomyelins	5.07 (4.4-6.4)	6.99 (5.91-7.86)	1.28	Increase	0.0000
SM (OH) C16:1	Sphingomyelins	1.47 (1.28-2.01)	2.11 (1.86-2.565)	1.36	Increase	0.0000
SM C20:2	Sphingomyelins	0.266 (0.182-0.357)	0.377 (0.2815-0.557)	1.49	Increase	0.0002
SM (OH) C24:1	Sphingomyelins	0.521 (0.419-0.664)	0.599 (0.526-0.685)	1.14	Increase	0.0275
SM C26:1	Sphingomyelins	0.161 (0.126-0.189)	0.174 (0.153-0.226)	1.13	Increase	0.0473
SM C26:0	Sphingomyelins	0.075 (0.063-0.097)	0.096 (0.079-0.1155)	1.21	Increase	0.0017
Cer(d18:1/24:1)	Ceramides	0.581 (0.488-0.76)	0.928 (0.6815-1.08)	1.45	Increase	0.0000
Cer(d18:1/24:0)	Ceramides	1.54 (1.17-1.86)	2.12 (1.685-2.465)	1.32	Increase	0.0007
Cer(d18:1/16:0)	Ceramides	0.291 (0.238-0.343)	0.434 (0.368-0.5555)	1.51	Increase	0.0000
Cer(d18:1/22:0)	Ceramides	0.556 (0.452-0.715)	0.757 (0.6405-0.9545)	1.37	Increase	0.0006
Cer(d18:1/23:0)	Ceramides	0.449 (0.365-0.599)	0.649 (0.5105-0.7895)	1.38	Increase	0.0002
Cer(d18:2/24:1)	Ceramides	0.13 (0.103-0.171)	0.188 (0.1555-0.2565)	1.43	Increase	0.0000
Cer(d18:1/25:0)	Ceramides	0.205 (0.169-0.259)	0.297 (0.2225-0.3415)	1.30	Increase	0.0004
Cer(d18:1/20:0)	Ceramides	0.1 (0.071-0.129)	0.154 (0.1105-0.1965)	1.60	Increase	0.0001
Cer(d18:2/16:0)	Ceramides	0.063 (0.051-0.076)	0.11 (0.082-0.1395)	1.67	Increase	0.0000

Cer(d18:2/22:0)	Ceramides	0.22 (0.162-0.262)	0.276 (0.1995-0.314)	1.24	Increase	0.0254
Cer(d18:2/23:0)	Ceramides	0.073 (0.059-0.101)	0.093 (0.0735-0.11)	1.23	Increase	0.0256
HexCer(d18:1/24:1)	Hexosylceramides	2.19 (1.71-2.94)	3.04 (2.395-3.89)	1.37	Increase	0.0012
HexCer(d18:1/16:0)	Hexosylceramides	0.665 (0.519-0.878)	1.14 (0.929-1.29)	1.50	Increase	0.0000
HexCer(d18:1/22:0)	Hexosylceramides	2.41 (1.86-3.23)	3.24 (2.395-4.01)	1.27	Increase	0.0069
HexCer(d18:1/23:0)	Hexosylceramides	0.868 (0.681-1.15)	1.21 (0.97-1.335)	1.31	Increase	0.0012
HexCer(d18:1/24:0)	Hexosylceramides	1.15 (0.967-1.6)	1.56 (1.24-1.81)	1.22	Increase	0.0103
HexCer(d18:2/24:0)	Hexosylceramides	0.696 (0.538-0.874)	0.955 (0.7435-1.065)	1.29	Increase	0.0047
HexCer(d18:1/20:0)	Hexosylceramides	0.173 (0.141-0.248)	0.274 (0.2215-0.3425)	1.48	Increase	0.0002
HexCer(d18:2/22:0)	Hexosylceramides	0.496 (0.369-0.643)	0.716 (0.456-0.844)	1.32	Increase	0.0107
Hex2Cer(d18:1/16:0)	Dihexosylceramides	1.9 (1.52-2.45)	2.95 (2.16-3.585)	1.47	Increase	0.0000
Hex2Cer(d18:1/24:1)	Dihexosylceramides	0.228 (0.168-0.294)	0.361 (0.255-0.453)	1.61	Increase	0.0000
Hex2Cer(d18:1/18:0)	Dihexosylceramides	0.101 (0.085-0.126)	0.138 (0.1275-0.169)	1.41	Increase	0.0000
Hex2Cer(d18:1/22:0)	Dihexosylceramides	0.133 (0.105-0.164)	0.182 (0.137-0.2555)	1.44	Increase	0.0005

Hex2Cer(d18:1/14:0)	Dihexosylceramides	0.082 (0.068-0.114)	0.121 (0.0935-0.147)	1.34	Increase	0.0017
Hex3Cer(d18:1/16:0)	Trihexosylceramides	0.868 (0.655-1.09)	1.45 (1.14-1.8)	1.59	Increase	0.0000
Hex3Cer(d18:1/24:1)	Trihexosylceramides	0.28 (0.198-0.334)	0.413 (0.3225-0.5485)	1.49	Increase	0.0000
Hex3Cer(d18:1_22:0)	Trihexosylceramides	0.255 (0.202-0.324)	0.43 (0.316-0.5015)	1.64	Increase	0.0000
PC aa C34:2	Phosphatidylcholines	254 (213-308)	304 (265.5-370.5)	1.19	Increase	0.0109
PC aa C36:2	Phosphatidylcholines	148 (122-179)	182 (142.5-210.5)	1.18	Increase	0.0093
PC aa C32:0	Phosphatidylcholines	8.28 (6.74-10.3)	12.9 (10.25-15.2)	1.45	Increase	0.0000
PC ae C38:5	Phosphatidylcholines	13.1 (11.8-15.3)	15.2 (13.4-18.1)	1.14	Increase	0.0080
PC ae C34:1	Phosphatidylcholines	4.41 (3.87-5.73)	5.95 (5.29-7.235)	1.27	Increase	0.0002
PC ae C38:4	Phosphatidylcholines	8.75 (7.57-10.6)	10.4 (8.715-12.3)	1.14	Increase	0.0108
PC ae C36:4	Phosphatidylcholines	12.6 (10.9-14.7)	14.9 (11.55-17.45)	1.17	Increase	0.0093
PC ae C34:2	Phosphatidylcholines	7.23 (6.01-8.57)	9.23 (7.69-11.65)	1.26	Increase	0.0021
PC ae C34:3	Phosphatidylcholines	5.55 (4.51-6.72)	7.46 (5.11-9.585)	1.30	Increase	0.0031
PC ae C36:2	Phosphatidylcholines	7.61 (6.59-9.56)	9.45 (7.925-10.95)	1.15	Increase	0.0162
PC ae C32:1	Phosphatidylcholines	1.54 (1.24-1.9)	2.18 (1.945-2.945)	1.49	Increase	0.0000

PC ae C36:5	Phosphatidylcholines	7.61 (6.58-8.77)	8.46 (7.475-10.4)	1.14	Increase	0.0306
PC ae C36:3	Phosphatidylcholines	4.49 (3.77-5.54)	5.73 (4.905-6.67)	1.19	Increase	0.0088
PC ae C40:6	Phosphatidylcholines	3.02 (2.54-3.61)	3.4 (3.065-3.97)	1.12	Increase	0.0175
PC aa C30:0	Phosphatidylcholines	1.19 (1.03-1.65)	1.77 (1.365-2.11)	1.33	Increase	0.0001
PC ae C36:1	Phosphatidylcholines	3.34 (2.82-4.26)	3.79 (3.265-4.425)	1.13	Increase	0.0358
PC ae C40:5	Phosphatidylcholines	1.92 (1.76-2.3)	2.15 (1.89-2.64)	1.12	Increase	0.0216
PC ae C44:5	Phosphatidylcholines	0.91 (0.752-1.11)	1.22 (0.8165-1.415)	1.21	Increase	0.0185
PC ae C44:6	Phosphatidylcholines	1.15 (0.975-1.4)	1.43 (1.105-1.6)	1.19	Increase	0.0104
PC ae C42:5	Phosphatidylcholines	1.22 (1.07-1.44)	1.48 (1.15-1.7)	1.14	Increase	0.0180
PC ae C40:4	Phosphatidylcholines	1.65 (1.45-1.86)	1.9 (1.605-2.215)	1.12	Increase	0.0366
PC ae C40:2	Phosphatidylcholines	0.768 (0.608-0.905)	0.883 (0.765-1.085)	1.20	Increase	0.0053
PC aa C40:4	Phosphatidylcholines	1.75 (1.48-2.16)	2.05 (1.76-2.635)	1.17	Increase	0.0185
PC aa C42:0	Phosphatidylcholines	0.414 (0.346-0.523)	0.567 (0.442-0.6695)	1.23	Increase	0.0044
PC ae C42:4	Phosphatidylcholines	0.639 (0.529-0.737)	0.703 (0.59-0.958)	1.18	Increase	0.0357

PC ae C34:0	Phosphatidylcholines	0.669 (0.523-0.78)	0.721 (0.6295-0.9995)	1.20	Increase	0.0382
PC ae C36:0	Phosphatidylcholines	0.412 (0.347-0.509)	0.533 (0.446-0.636)	1.27	Increase	0.0006
PC ae C32:2	Phosphatidylcholines	0.381 (0.307-0.475)	0.454 (0.4025-0.5875)	1.24	Increase	0.0032
PC aa C40:3	Phosphatidylcholines	0.322 (0.251-0.389)	0.398 (0.3265-0.484)	1.26	Increase	0.0011
PC aa C42:1	Phosphatidylcholines	0.267 (0.215-0.326)	0.328 (0.2625-0.37)	1.18	Increase	0.0188
PC ae C42:2	Phosphatidylcholines	0.272 (0.224-0.322)	0.33 (0.2885-0.399)	1.20	Increase	0.0032
PC aa C32:3	Phosphatidylcholines	0.324 (0.262-0.38)	0.37 (0.339-0.427)	1.16	Increase	0.0074
PC ae C44:4	Phosphatidylcholines	0.239 (0.209-0.296)	0.282 (0.225-0.337)	1.16	Increase	0.0386
PC ae C30:2	Phosphatidylcholines	0.05 (0.042-0.059)	0.063 (0.056-0.0715)	1.20	Increase	0.0001
lysoPC a C18:2	Lysophosphatidylcholines	26.6 (22.6-31.6)	23.6 (19.2-27.5)	0.90	Decrease	0.0397
lysoPC a C20:3	Lysophosphatidylcholines	1.11 (0.962-1.33)	0.942 (0.804-1.1)	0.86	Decrease	0.0301
Glutamine	Amino acids	732 (652-795)	819 (744-897.5)	1.15	Increase	0.0002
Glycine	Amino acids	362 (329-420)	425 (378.5-474.5)	1.13	Increase	0.0033
Phenylalanine	Amino acids	93.2 (86.4-103)	109 (99.6-118.5)	1.18	Increase	0.0000
Glutamate	Amino acids	95 (85.9-110)	119 (103.5-127.5)	1.15	Increase	0.0033

Aspartate	Amino acids	33.7 (29.7-41.8)	46.7 (37.45-52.9)	1.36	Increase	0.0001
Arginine	Amino acids	140 (119-161)	162 (136.5-174.5)	1.11	Increase	0.0295
Cysteine	Amino acids	65.1 (57.3-73.3)	80.5 (71.75-91.95)	1.23	Increase	0.0000
Sarcosine	Amino acid related	1.68 (1.39-1.95)	1.92 (1.58-2.24)	1.13	Increase	0.0492
Taurine	Amino acid related	77.1 (68.6-91.4)	88.2 (77.6-99.85)	1.13	Increase	0.0071
Creatinine	Amino acid related	78.7 (65.6-92.1)	101 (74.35-121)	1.23	Increase	0.0075
Kynurenine	Amino acid related	2.07 (1.7-2.4)	2.54 (2.075-3.045)	1.27	Increase	0.0004
Asymmetric dimethylarginine	Amino acid related	0.581 (0.528-0.639)	0.651 (0.5965-0.7085)	1.11	Increase	0.0011
Symmetric dimethylarginine	Amino acid related	0.714 (0.588-0.911)	0.892 (0.665-1.205)	1.19	Increase	0.0199
Homocysteine	Amino acid related	9.53 (7.65-12.2)	11.7 (9.39-14.75)	1.08	Increase	0.0109
Betaine	Amino acid related	60.6 (49.1-75.3)	77.9 (64.95-90.45)	1.26	Increase	0.0005
Proline betaine	Amino acid related	5.78 (2.42-9.81)	8.78 (4.33-15.85)	1.85	Increase	0.0251
1-Methylhistidine	Amino acid related	5.1 (4.23-6.05)	6.84 (4.87-8.975)	1.41	Increase	0.0047
Methionine sulfoxide	Amino acid related	1.15 (0.91-1.39)	1.37 (1.185-1.49)	1.26	Increase	0.0047

Dihydroxyphenylalanine	Amino acid related	0.038 (0.032-0.052)	0.113 (0.1115-0.1195)	2.05	Increase	0.0000
β-Aminobutyric acid	Amino acid related	0.037 (0.029-0.045)	0.057 (0.0395-0.088)	1.62	Increase	0.0003
Anserine	Amino acid related	0.002 (0.001-0.003)	0.005 (0.004-0.007)	1.96	Increase	0.0000
Cystine	Amino acid related	66 (55.3-79.5)	105 (87.95-120)	1.55	Increase	0.0000
Citrulline	Amino acid related	45.5 (36.7-53.5)	54.5 (43.35-67.05)	1.17	Increase	0.0138
Carnosine	Amino acid related	0.037 (0.035-0.038)	0.025 (0.023-0.026)	0.72	Decrease	0.0000
Phenylacetylglycine	Amino acid related	0.004 (0.002-0.006)	0.003 (0.002-0.0045)	0.70	Decrease	0.0278
Dopamine	Biogenic amines	0.079 (0.076-0.082)	0.261 (0.26-0.262)	2.20	Increase	0.0000
γ-Aminobutyric acid	Biogenic amines	0.153 (0.122-0.181)	0.227 (0.2005-0.2605)	1.53	Increase	0.0000
Histamine	Biogenic amines	0.074 (0.072-0.08)	0.114 (0.113-0.116)	1.37	Increase	0.0000
Spermine	Biogenic amines	0.154 (0.144-0.167)	0.181 (0.165-0.202)	1.14	Increase	0.0000
Spermidine	Biogenic amines	0.207 (0.167-0.236)	0.258 (0.212-0.286)	1.18	Increase	0.0017
Putrescine	Biogenic amines	0.118 (0.098-0.154)	0.149 (0.121-0.1745)	1.14	Increase	0.0045
Aconitic acid	Carboxylic acids	0.687 (0.579-0.877)	1.24 (1.12-1.595)	1.75	Increase	0.0000
Dodecanedioic acid	Carboxylic acids	0.017 (0.012-0.02)	0.02 (0.014-0.032)	2.17	Increase	0.0363

Tetradecanedioic acid	Carboxylic acids	0.023 (0.017-0.035)	0.038 (0.028-0.0455)	1.47	Increase	0.0002
Succinic acid	Carboxylic acids	1.53 (1.39-1.74)	1.33 (1.115-1.495)	0.99	Decrease	0.0013
Acetylcarnitine	Acylcarnitines	4.77 (4.17-6.01)	6.82 (6.135-7.955)	1.36	Increase	0.0000
Deoxycholic acid	Bile acids	6.07 (5.29-7.17)	7.31 (6.795-7.855)	1.16	Increase	0.0003
Taurocholic acid	Bile acids	0.018 (0.009-0.061)	0.063 (0.0535-0.1345)	2.41	Increase	0.0000
Glycocholic acid	Bile acids	0.185 (0.098-0.369)	0.306 (0.161-0.727)	1.60	Increase	0.0109
Choline	Vitamins and cofactors	13.5 (12.4-16.1)	17.3 (14.9-19.2)	1.22	Increase	0.0000
Hypoxanthine	Nucleobases and related	21.8 (19.9-25.2)	19.4 (16.2-22.8)	0.95	Decrease	0.0170

Abbreviation: TG - triglyceride; DG - diglyceride; FA - fatty acid; CE - cholesteryl ester; SM - sphingomyelin; Cer - ceramide; Hexcer - hexosylceramide; Hex2Cer - dihexosylceramide; Hex3Cer-trihexosylceramide; PCs - phosphatidylcholine; LysoPCs - lysophosphatidylcholine; FDR-false discovery rate;

Table S3. Metabolites with FDR<0.05 and VIP>1.5

Metabolites	Categories	Serum Concentration (umol/L)		Foldchange	FDR	VIP
		Stage B (n=97)	Stage C (n=31)			
FA(18:0)	Fatty acids	187 (161-209)	289 (229.5-315)	1.46	<0.001	5.19
Glutamine	Amino acids	732 (652-795)	819 (744-897.5)	1.15	<0.001	4.72
TG(16:0_36:3)	Triglycerides	159 (116-246)	252 (189-351)	1.63	<0.001	4.17
TG(18:1_34:2)	Triglycerides	152 (115-231)	249 (174-347)	1.63	<0.001	4.13
TG(18:2_34:1)	Triglycerides	154 (113-236)	245 (175-327.5)	1.61	<0.001	3.98
TG(18:2_34:2)	Triglycerides	161 (109-222)	260 (161-318.5)	1.62	<0.001	3.89
FA(16:0)	Fatty acids	299 (270-325)	345 (322.5-386.5)	1.18	<0.001	3.72
TG(18:1_36:3)	Triglycerides	114 (90.1-174)	178 (121-258)	1.55	0.002	3.44
SM C16:0	Sphingomyelins	74.9 (63.4-90.3)	118 (103.5-146.5)	1.52	<0.001	3.20
Cystine	Amino acid related	66 (55.3-79.5)	105 (87.95-120)	1.55	<0.001	3.19
TG(16:0_36:4)	Triglycerides	89.9 (61.4-129)	143 (94.35-178.5)	1.63	<0.001	2.91

CE(20:4)	Cholesteryl esters	242 (186-319)	307 (246.5-374.5)	1.26	0.010	2.89
TG(18:2_36:2)	Triglycerides	72.9 (53.9-107)	118 (82.2-162)	1.58	0.001	2.79
TG(18:2_36:3)	Triglycerides	85 (58.8-111)	109 (82.35-170)	1.56	0.007	2.74
CE(16:0)	Cholesteryl esters	155 (122-214)	216 (188.5-276)	1.34	<0.001	2.72
FA(18:2)	Fatty acids	179 (140-214)	215 (177.5-246)	1.23	0.005	2.36
Glycine	Amino acids	362 (329-420)	425 (378.5-474.5)	1.13	0.003	2.29
PC aa C34:2	Phosphatidylcholines	254 (213-308)	304 (265.5-370.5)	1.19	0.011	2.17
TG(18:1_36:4)	Triglycerides	55.5 (38.1-72.8)	73.7 (53-113.5)	1.52	0.011	2.13
Creatinine	Amino acid related	78.7 (65.6-92.1)	101 (74.35-121)	1.23	0.008	2.00
Phenylalanine	Amino acids	93.2 (86.4-103)	109 (99.6-118.5)	1.18	<0.001	1.93
Cysteine	Amino acids	65.1 (57.3-73.3)	80.5 (71.75-91.95)	1.23	<0.001	1.91
FA(18:1)	Fatty acids	123 (97.9-149)	136 (122.5-163)	1.18	0.013	1.83
Glutamate	Amino acids	95 (85.9-110)	119 (103.5-127.5)	1.15	0.003	1.79
Aspartate	Amino acids	33.7 (29.7-41.8)	46.7 (37.45-52.9)	1.36	<0.001	1.71

Betaine	Amino acid related	60.6 (49.1-75.3)	77.9 (64.95-90.45)	1.26	0.001	1.59
TG(18:2_36:4)	Triglycerides	32.4 (20.8-45.7)	42.6 (27.3-71.05)	1.53	0.023	1.53
PC aa C36:2	Phosphatidylcholines	148 (122-179)	182 (142.5-210.5)	1.18	0.009	1.51

Abbreviation: FA, fatty acid; TG, triglyceride; CE, cholesteryl ester; SM, sphingomyelin; PCs, phosphatidylcholine; FDR, false discovery rate; VIP, variable importance in projection.
